# Environmental Responses and Interspecific Associations of Fish Communities in the Zhoushan Fishing Ground Revealed by HMSC

**DOI:** 10.3390/ani16060865

**Published:** 2026-03-10

**Authors:** Xiaoyan Mao, Jing Wang, Yang Liu, Hui Ge, Haichen Zhu, Yongdong Zhou, Hongliang Zhang, Mingyang Xie, Wenbin Zhu

**Affiliations:** 1 College of Fishery, Zhejiang Ocean University, Zhoushan 316022, China; maoxiaoyan830@163.com (X.M.); wangjing@zjou.edu.cn (J.W.); liuyang20000831@163.com (Y.L.); 20250084@zjou.edu.cn (M.X.); 2 Zhejiang Marine Fisheries Research Institute, Zhoushan 316021, China; zyd511@126.com (Y.Z.); hidalgo310@163.com (H.Z.); 3 Jiangsu Marine Fisheries Research Institute, Nantong 226007, China; gehui_xi@163.com (H.G.); hajsen@126.com (H.Z.)

**Keywords:** Zhoushan Fishing Ground, fish community, joint species distribution modeling, environmental responses, interspecific associations

## Abstract

The fish community structure of the Zhoushan Fishing Ground is undergoing change due to overfishing, climate variability, and other anthropogenic stressors. To investigate community-level environmental responses and interspecific associations in this region, we used 11 consecutive years (2014–2024) of spring bottom trawl survey data from the Zhoushan Fishing Ground and integrated environmental covariates to build a two-part hurdle model within the Hierarchical Modelling of Species Communities (HMSC) framework. The results showed that the spatial random effect had the highest contribution (41%), followed by the interannual trend (18%), indicating that community occurrence patterns are primarily shaped by the superposition of stable spatial structuring and long-term change. Depth was significant for more species, whereas salinity was significant for the fewest. Residual correlations further revealed that the focal fish species could be partitioned into two assemblages with one linking species. Meanwhile, within the two-part hurdle model, the direction and significance of responses to the same covariate were not always consistent, supporting that species occurrence probability and positive biomass are governed by different ecological processes. Overall, this study provides a transferable quantitative framework for community assessment in coastal fishing grounds and offers a more operational chain of evidence for ecosystem-based fisheries management.

## Simple Summary

The Zhoushan Fishing Ground is the largest fishing ground in China, and its fish community structure is currently undergoing change under the influence of multiple factors. To gain a deeper understanding of the fish community in this region, we built models based on 11 consecutive years of data from 2014 to 2024 for analysis. The results showed that fish distribution patterns were mainly associated with spatial differences among areas and long-term trends. In addition, the focal fish species examined in this study could be broadly divided into two groups, with one species showing associations with both groups. Meanwhile, we found that the drivers of species occurrence and those influencing positive biomass were not identical. These findings not only deepen our understanding of fish communities in the Zhoushan Fishing Ground, but also provide reference information for the conservation and management of fisheries resources, thereby helping to promote their sustainable use.

## 1. Introduction

Driven by climate-induced environmental change and human activities, the stability of marine fisheries ecosystems is being challenged [1,2,3]. As an important component of marine ecosystems, fishes exhibit distributions and community structures that are highly sensitive to environmental change, thereby reflecting the evolution of the marine environment [3,4]. Meanwhile, changes in fish community structure directly affect the stability and sustainability of fisheries resource utilization [5]. For example, juvenile fish are highly sensitive to environmental conditions in spawning and nursery habitats and to transport processes shaped by currents and salinity gradients [6]. If environmental change impedes the replenishment process and reduces the number of juveniles entering adult populations, adult stock size may show a decline or increased variability [6]. This reduction and fluctuation in adult resources can further influence community structure and catch stability, thereby undermining the sustainability of fisheries resource use [3,7]. Therefore, analyzing the spatiotemporal patterns and dynamics of fisheries resources at the fish community level has become an important research direction in fisheries ecology and fisheries resource management [7,8].

Traditional species distribution models (SDMs) typically assume that species respond independently to changes along environmental gradients [9]. However, in natural ecosystems, species often exhibit coordinated responses to environmental change, rather than responding independently, due to factors such as niche similarity and biotic interactions [10,11]. Single-species models often overlook interspecific correlations, limiting their ability to capture joint responses to environmental gradients [12]. Consequently, such approaches fail to reveal the overarching ecological patterns underlying changes in community structure and are therefore limited in explaining community dynamics [12,13].

To address the limitations of single-species SDMs, joint species distribution models (JSDMs) have been developed. They can analyze multiple species simultaneously, enabling concurrent estimation of the effects of environmental factors and residual associations among species [14]. These associations may indicate interspecific interactions and may also reflect shared responses to unobserved environmental variables [15]. Accordingly, JSDMs provide a more integrative and efficient approach for revealing community-level dynamics [16]. Within the JSDM family, the Hierarchical Modelling of Species Communities (HMSC) framework is grounded in community assembly theory and places species’ responses to environmental gradients within a hierarchical structure [17]. This enables the extraction of shared community-level signals across species while characterizing interspecific differences, which is beneficial for identifying medium- to long-term change patterns in multi-year time series data [18]. Moreover, because it adopts hierarchical modeling, HMSC can incorporate, within a unified framework, the stratified structure implicit in survey design together with multi-scale spatiotemporal context, thereby supporting the partitioning of sources of variation in community patterns [19]. Specifically, HMSC can include stratified sampling units and spatiotemporal context (e.g., sites and years) as random effects in joint modeling, and within a unified inferential framework, it can characterize environmental filtering and spatiotemporal structure, while estimating residual species associations after accounting for the environmental covariates, thereby enabling the relative contributions of these processes to community variation to be quantified and compared [17,20]. To date, HMSC has been applied in community research and management contexts in both marine and terrestrial systems [21,22].

The Zhoushan Fishing Ground is located east of Hangzhou Bay and is influenced by diluted freshwater plumes from the Qiantang River, the Yangtze River, the Yong River, and other rivers. The region contains numerous islands and features distinctive geographic and hydrological conditions as well as a complex fisheries ecosystem structure. It is the largest fishing ground in China. In recent years, driven by pollution, overfishing, and environmental change, a range of issues has gradually emerged, including catch miniaturization, a decline in the mean trophic level of catches, and reduced yields of commercially important species [23]. The composition of fishery resources and the fish community structure in this region have changed. However, existing studies have mainly focused on single species or short time series. Consequently, quantitative evidence based on long-term time series that allows direct community-level comparisons is still lacking regarding the spatiotemporal dynamic patterns of fish communities in the Zhoushan Fishing Ground over longer time scales, the roles of key environmental factors, and whether stable co-occurrence structure and spatial zonation exist among species. Based on the above background, we integrated spring bottom-trawl survey data and environmental variables from the Zhoushan Fishing Ground collected over 11 consecutive years (2014–2024), and conducted joint modeling of the fish community within the HMSC framework. Specifically, we addressed the following questions and hypotheses: (1) What are the relative contributions of spatiotemporal patterns and environmental factors to fish distributions? Hypothesis 1. Fish distributions in the Zhoushan Fishing Ground are primarily governed by spatial differences between areas and long-term temporal trends, whereas environmental covariates (temperature, salinity, depth, and chlorophyll-a) play a secondary but species-specific role. (2) After accounting for environmental differences, does the community exhibit stable spatial organization and species assemblages? Hypothesis 2. After accounting for environmental differences, the fish community exhibits stable spatial organization and can be partitioned into several stable species assemblages, with predominantly positive associations within assemblages and predominantly negative associations between assemblages. (3) Are distribution and resource abundance governed by different ecological processes and therefore show different responses to external pressures? Hypothesis 3. Species occurrence (distribution) and positive biomass (resource abundance) are governed by different ecological processes, and their responses to the same environmental covariates (external pressures) can differ. By quantifying the effects of spatiotemporal structure and key environmental factors within a unified framework, further elucidating community species assemblages and spatial organization, and distinguishing information on distribution and resource abundance, this study provides evidence to support fisheries management at both the “distribution” and “resource abundance” levels. These findings help identify key habitats and areas of high resource abundance, supporting spatial zoning and multispecies stock assessments.

## 2. Materials and Methods

### 2.1. Data Sources

**Figure 1:**
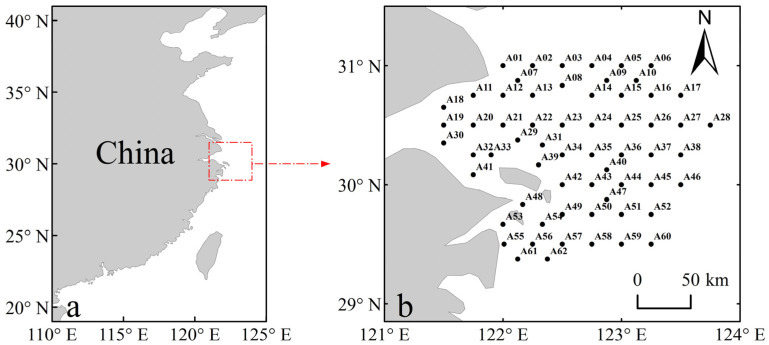
(**a**) Location of the study area in China; (**b**) Location of sampling stations in the Zhoushan Fishing Ground.

The species biomass data used in this study were obtained from the fisheries resources survey program in the Zhoushan Fishing Ground. Surveys were conducted each April from 2014 to 2024. We selected spring survey data because spring is a critical period for the reproduction and recruitment of many coastal fish species. Meanwhile, restricting the survey to April each year helps reduce seasonal variability when assessing interannual changes. The survey area covered 29°00′ N–31°30′ N and 121°00′ E–124°00′ E, and a total of 62 sampling stations were established within the study region (Figure 1). Bottom trawl surveys were conducted using bottom trawl vessels equipped with trawl gear (net mouth perimeter: 50 m; codend mesh size: 25 mm). Given the codend mesh size, the survey may underestimate smaller juveniles or larvae; therefore, our inferences primarily pertain to the fisheries resources that can be effectively sampled by the bottom-trawl survey. At each station, one haul was conducted per year using an identical gear configuration, and the net was towed at a speed of 3 kn for 1 h. All procedures, including sampling, sample processing, and biological measurements, were conducted with reference to the Specifications for oceanographic survey–Part 6: Marine biological survey (GB/T 12763.6–2007) [24] and the Technical specification for marine fishery resources survey (SC/T 9403–2012) [25]. Environmental variables included sea surface temperature (SST), sea surface salinity (SSS), chlorophyll a (Chl–a), and water depth (Depth). SST, SSS, and Chl–a were obtained from the Copernicus Marine Data Store (see Table S1 for details) [26], whereas Depth was derived from the General Bathymetric Chart of the Oceans (GEBCO) [27]. Environmental data were extracted from gridded datasets using nearest-neighbor interpolation, ensuring spatial alignment with species biomass records.

In this study, we incorporated a subset of surface environmental variables to explain the fish distribution derived from bottom trawl surveys, to characterize regional scale water mass properties and the productivity background. The study area is strongly influenced by current–topography interactions and intense tidal mixing, which can enhance vertical mixing and weaken water column stratification, leading to a more vertically homogeneous water column and thereby increasing the extent to which surface signals reflect the regional hydrographic background [28]. Therefore, using surface variables as explanatory predictors of regional environmental conditions is justified. Nevertheless, surface variables cannot fully represent bottom environmental conditions, and the associated results should be interpreted primarily as responses to broad scale environmental gradients. To better account for habitat constraints, Depth was further included as an indirect proxy for bottom environmental conditions [29]. Combined with the surface background variables, it provides a more comprehensive characterization of the major environmental gradients shaping the distribution of fish caught by bottom trawl surveys.

Because the dataset comprised survey observations from 62 sampling stations over 11 years, we preprocessed and filtered the data prior to modeling to mitigate the effects of zero inflation and rare species, thereby improving the stability of model fitting and the reliability of parameter estimation [30]. Based on station–year survey records, a species was considered present at a given station in a given year when its biomass was >0; otherwise, it was treated as absent. To ensure the statistical robustness of parameter estimation and inference and to avoid information scarcity and unstable estimates caused by rare species, we applied occurrence-based inclusion thresholds. Based on the number of years, the number of stations, and the total number of records in which each species occurred during the entire survey period, corresponding thresholds were applied (occurring in more than 5 years, at more than 30 stations, and with more than 50 occurrence records) to ensure that the species ultimately included in the model exhibited stable spatiotemporal distribution characteristics and provided sufficient information. Through this screening procedure, 26 fish species were retained for model construction (Table S2). Meanwhile, to facilitate species-level interpretation of interspecific heterogeneity in responses to environmental covariates and random effects, as well as species associations, we applied stricter screening criteria for visualization, while keeping the set of species included in model fitting unchanged. Specifically, focal species were required to be detected in all survey years, to occur at more than 42 stations, and to have more than 200 occurrence records to ensure sufficient temporal and spatial coverage and adequate sample size for stable and comparable visualization and species level inference. Using these criteria, we finally retained 12 focal fish species (Table 1).

### 2.2. Model Construction and Variable Selection

To accommodate the high frequency of zeros in bottom trawl survey data and to explicitly assess whether species occurrence probability and positive biomass are governed by different ecological processes, we used the HMSC framework to conduct two-part hurdle model joint modeling of multispecies data [31]. In the first part, species presence–absence was used as the response variable and modeled using a binomial distribution with a probit link (the PA model). In the second part, the POSBIO model was fitted using only observations with biomass > 0, and biomass was log1p-transformed. Fixed effect variables were selected to jointly represent the influences of the physical environment and primary productivity, including SST, SSS, Chl–a, and Depth. To capture potential nonlinear responses of fishes to temperature, based on empirical evidence from previous studies, a quadratic temperature term (SST^2^) was included [21]. Chl–a was log-transformed to improve its distributional properties, and all environmental variables were standardized prior to modeling to enhance the stability and comparability of parameter estimates. To describe potential long-term trends in the fish community during the study period, survey year was converted into a continuous variable (YearNum) and included as a fixed effect [32]. For random effects, site was specified as a spatial random effect and year as an independent and identically distributed (IID) temporal random effect; the former reflects spatial autocorrelation not explained by the environmental covariates, whereas the latter captures random interannual fluctuations [33].

### 2.3. Model Fitting and Diagnostics

During model fitting, we ran four mutually independent Markov chain Monte Carlo (MCMC) chains. For each chain, 3000 burn-in iterations were performed first, followed by posterior sampling with one sample retained every five iterations, yielding 3000 posterior draws. The total number of iterations per chain was 18,000, resulting in 3000 approximately independent posterior samples per chain and a total of 12,000 posterior draws across the four chains. Because HMSC involves a high-dimensional parameter space and strong posterior dependence, MCMC chains may mix slowly and exhibit substantial autocorrelation; therefore, model convergence and sampling efficiency were evaluated using the potential scale reduction factor (PSRF) and the effective sample size (ESS) [34,35]. When the mean PSRF was ≤1.1, MCMC convergence was considered satisfactory. Larger ESS values indicate weaker autocorrelation and higher sampling efficiency. Model performance was assessed from two aspects: in-sample explanatory performance and out-of-sample predictive performance [19]. The former was calculated based on posterior predictions from the full dataset, whereas the latter was obtained via fivefold cross validation to balance robustness and computational efficiency. The explanatory and predictive performance of the PA model were evaluated using the area under the receiver operating characteristic curve (AUC) and Tjur’s R^2^. AUC quantifies the model’s discriminatory ability to distinguish species presence from absence, where 0.5 indicates no discrimination, 0.7–0.8 is acceptable, 0.8–0.9 is excellent, and >0.9 is outstanding [36]. Tjur’s R^2^ represents the difference between the mean predicted probabilities for presence and absence observations; it is not an explanatory power metric in the traditional sense, and overly high values may also indicate overfitting to the training data [37]. Given that the POSBIO model has a continuous response variable, model performance was evaluated using the root mean square error (RMSE) and the coefficient of determination (R^2^). Smaller RMSE values indicate lower fitting and prediction errors, whereas larger R^2^ values indicate stronger explanatory power [21].

### 2.4. Model Analysis

Within the HMSC framework, species–environment responses were quantified by regression coefficients (β). When the posterior probability that β was positive (or negative) was ≥0.95, the response of a given species to that environmental factor was considered to have strong support. We then summarized the direction and strength of β for each predictor across the 12 focal fish species and visualized the results using a heatmap. To examine residual interspecific co-occurrence structure after accounting for environmental effects, we extracted the residual species association matrix (Ω) associated with the site-level random effect and expressed it as a correlation matrix for interpretation and visualization. To illustrate how species–environment relationships differ between the species occurrence probability (PA) and positive biomass (POSBIO) components of the two-part hurdle framework, we extracted posterior summaries of the regression coefficients (β) from the PA and POSBIO models and compared them side by side in a single figure. All analyses were performed in R 4.5.2, and the HMSC models were fitted using the Hmsc package (v3.3-7) [19]. The sampling station map was produced in ArcMap 10.8.

## 3. Results

### 3.1. Model Convergence Diagnostics and Performance Evaluation

Convergence diagnostics indicated that both the PA and POSBIO models converged well. The mean PSRF for fixed effect (β) and random effect (γ) parameters was close to 1 and well below the conventional threshold of 1.1 (Table 2). In addition, ESS were high, indicating low autocorrelation in the MCMC samples and efficient posterior sampling (Table 2). In terms of model performance, the PA model achieved an in-sample mean AUC of 0.84 and a mean Tjur’s R^2^ of 0.30. Under fivefold cross validation, the out-of-sample mean AUC was 0.81 and Tjur’s R^2^ was 0.28 (Table 2). The minimal difference between in-sample and out-of-sample performance indicates relatively consistent discriminatory ability and no evidence of overfitting. The POSBIO model yielded an in-sample RMSE of 1.04 and an R^2^ of 0.25, whereas under fivefold cross validation the out-of-sample RMSE was 1.11 and R^2^ was 0.07 (Table 2). The out-of-sample RMSE increased only slightly, whereas R^2^ decreased markedly, indicating relatively weak out-of-sample predictive performance for positive biomass magnitude.

### 3.2. Overall Variance Contributions and Differences Across Factors in the PA Model

**Figure 2:**
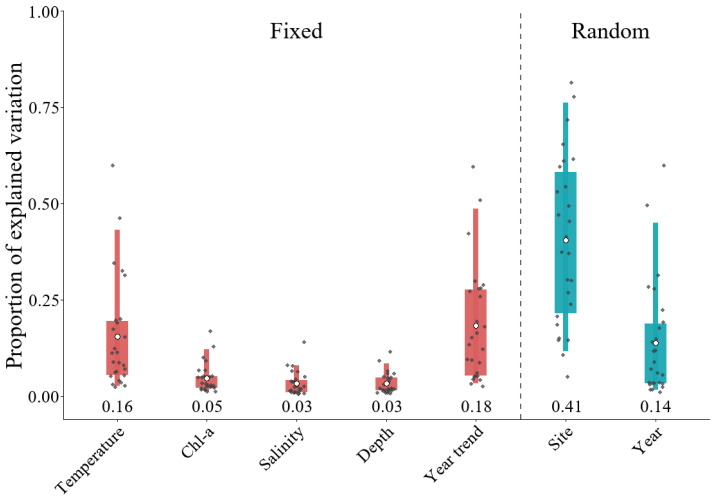
Variance partitioning of fixed and random effects in the PA model: dominant spatial effects. In all figures, “Temperature” represents the combined contribution of SST and SST^2^, and “Salinity” corresponds to SSS. Grey dots represent species-level variance contributions (each dot corresponds to one species). Thick line segments indicate the interquartile range (25th–75th percentiles), whereas thin line segments denote the 5th–95th percentile range. Open circles denote the mean contribution of each factor, and the numbers displayed above the x-axis report the corresponding mean values.

The contributions of different fixed and random effects to variation in fish occurrence probability differed markedly. Fixed and random effects explained 45% and 55% of the variation, respectively. Among these components, the spatial random effect accounted for the largest share of variation in species occurrence (41%), followed by the interannual trend (18%), temperature (SST and SST^2^; 16%), and the year random effect (14%). Finally, Chl–a, SSS, and Depth explained 5%, 3%, and 3% of the variation, respectively (Figure 2). At the species level, the relative contributions of explanatory factors varied substantially among species, with the same factor explaining different proportions of variation across species (Figure 2).

### 3.3. Sources of Variation and Differential Environmental Responses in Focal Species’ Occurrence Probability in the PA Model

**Figure 3:**
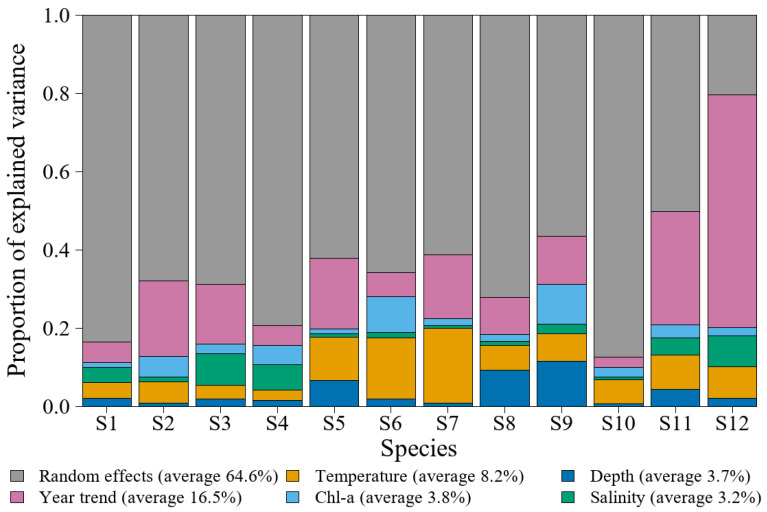
Variance partitioning of occurrence probability for the 12 focal fish species in the PA model. Species codes S1–S12 are consistent across all figures and correspond to those listed in Table 1.

Sources of variation differed substantially among species in the PA model. Overall, random effects were dominant on average (64.6%), followed by the year trend (16.5%) and temperature (SST and SST^2^; 8.2%), whereas the overall contributions of Chl–a (3.8%), Depth (3.7%), and salinity (3.2%) were relatively low (Figure 3). At the species level, in contrast to the other species, *Cynoglossus robustus* exhibited a relatively low contribution of random effects (20.4%), with the year trend instead accounting for the largest proportion of variation (59.5%; Figure 3; Table S3). For *Miichthys miiuy*, the contributions of fixed effects (49.7%) and random effects (50.3%) were broadly comparable, and the year trend also contributed relatively strongly (28.8%; Figure 3; Table S3), whereas random effects dominated for the remaining species (Figure 3).

**Figure 4:**
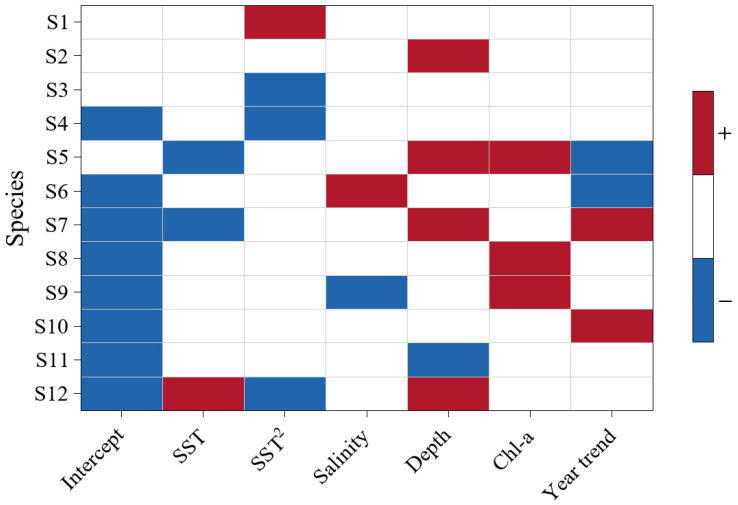
Direction and significance of focal fish species responses to environmental covariates in the PA model. Red indicates a positive relationship and blue indicates a negative relationship; colors are shown only when the posterior probability is ≥0.95, and blank cells indicate no strong support.

The PA model results indicated pronounced species specificity in significant responses of occurrence probability to environmental covariates. Marked interspecific differences were observed in significant responses to the linear temperature term (SST) and the quadratic term (SST^2^). Only *Cynoglossus robustus* showed significant responses to both SST and SST^2^, with a positive effect of SST and a negative effect of SST^2^ (Figure 4). Except for *Cynoglossus robustus*, only *Larimichthys polyactis* and *Lophius litulon* responded significantly to SST, whereas several species showed significance only for SST^2^. Among all fixed effects, depth was significantly associated with occurrence probability for the largest number of species (5/12), whereas salinity was significant for the fewest (2/12; Figure 4).

### 3.4. Residual Association Structure and Assemblage Partitioning of Focal Fish Species in the PA Model

**Figure 5:**
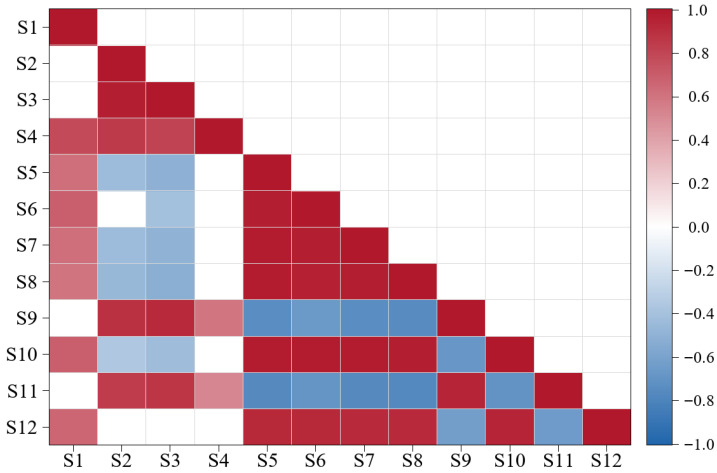
Residual correlation structure of focal fish species in the PA model, revealing two assemblages and a bridging species. Red indicates positive correlations and blue indicates negative correlations, with darker colors representing stronger correlation strength; only associations with posterior support ≥ 0.95 are shown, whereas blank cells indicate associations lacking strong support.

As shown in Figure 5, the 12 focal fish species could be identified as two assemblages, with one species exhibiting connecting characteristics. Specifically, strong positive residual correlations were observed among *Larimichthys polyactis*, *Chelidonichthys kumu*, *Lophius litulon*, *Erisphex pottii*, *Thryssa kammalensis*, and *Cynoglossus robustus*, forming a tightly associated assemblage (Assemblage 1). Assemblage 2 was composed of *Cynoglossus lighti*, *Coilia mystus*, *Ctenotrypauchen chinensis*, *Collichthys lucidus*, and *Miichthys miiuy*, with relatively strong positive residual correlations within the assemblage. Residual correlations between the two assemblages were predominantly negative. *Harpadon nehereus* showed relatively weak positive residual correlations with species in Assemblage 1 and also exhibited a weak positive residual correlation with *Collichthys lucidus* in Assemblage 2, suggesting its potential bridging role between the two assemblages.

### 3.5. Comparison of Environmental Responses of Focal Fish Species Between the PA and POSBIO Models

**Figure 6:**
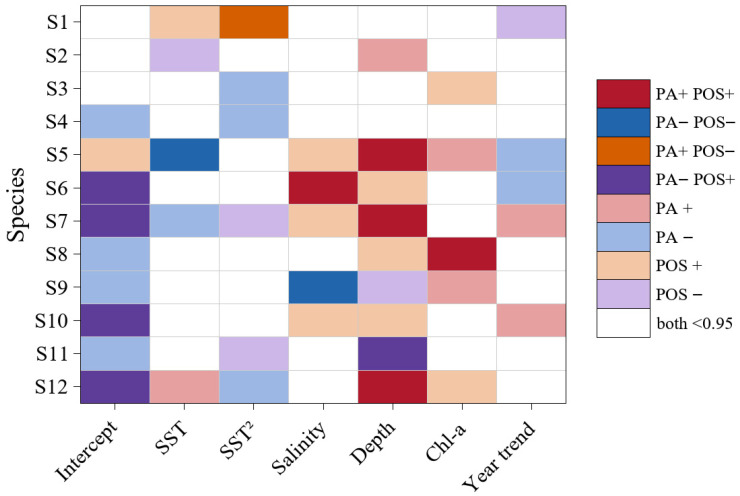
Comparison of response directions and significance of environmental effects for focal fish species between the PA and POSBIO models. In the legend, + indicates a positive correlation, and − indicates a negative correlation.

The focal fish species showed marked differences in the direction and significance of their responses to environmental covariates between the two models. Cases in which responses were significant in both models and consistent in direction were relatively rare. Consistent significant negative correlations occurred only for *Larimichthys polyactis* with SST and for *Ctenotrypauchen chinensis* with salinity. Consistent significant positive correlations were mainly associated with Depth, including significant positive relationships with Depth for *Larimichthys polyactis*, *Lophius litulon*, and *Cynoglossus robustus* (Figure 6). In addition, *Chelidonichthys kumu* showed a consistent significant positive association with salinity, and *Erisphex pottii* showed a consistent significant positive association with Chl–a. Notably, *Harpadon nehereus* was significantly positively associated with SST^2^ in the PA model but significantly negatively associated with SST^2^ in the POSBIO model, whereas *Miichthys miiuy* was significantly positively associated with depth in the POSBIO model but significantly negatively associated with depth in the PA model (Figure 6).

## 4. Discussion

### 4.1. Spatiotemporal Structure and Environmental Factors Jointly Shape Fish Distributions

The sources of variation in fish occurrence probability in the Zhoushan Fishing Ground exhibited a pattern of spatial structure dominance with concurrent interannual trends. This indicates that fish occurrence probability in the Zhoushan Fishing Ground is mainly influenced by spatial structuring processes captured by the spatial random effect and by overall temporal processes at the interannual scale (i.e., the year trend and the year random effect) [2,18,38]. The Zhoushan Fishing Ground is jointly influenced by estuarine diluted water, coastal currents, and offshore warm currents, resulting in complex water mass structure, frequent frontal activity, and pronounced spatial heterogeneity in the region [39]. Previous studies have shown that fronts and water mass boundaries in this area can lead to spatial differences in fish community distributions, thereby producing a certain pattern of spatial zonation [40]. Accordingly, the dominance of the spatial random effect in this study suggests that, beyond the environmental covariates already included in the model, unquantified spatially structured factors play an important role in shaping fish community patterns [20,41]. These factors may include small-scale hydrodynamic conditions, habitat heterogeneity, and sediment type. From a management perspective, this result indicates that the fishing ground exhibits a pronounced and relatively stable spatial structure, and thus spatial zoning and habitat-oriented management should be prioritized. In addition to the spatial random effect, the interannual trend accounted for the largest proportion of variation, exceeding that of all environmental covariates, indicating that the community exhibits a coherent long-term trend. This is consistent with findings on the long-term evolution of fish communities in the East China Sea, where the Zhoushan Fishing Ground is located. Previous studies have reported a fishing down (FD) phenomenon in this region, whereby the mean trophic level of catches shows a long-term decline and fishing targets shift from large, high-trophic level species to small, low-trophic level species, suggesting that sustained fishing pressure may influence long-term community dynamics [23]. Other studies have also indicated that under high-intensity fishing pressure, the East China Sea ecosystem may experience structural community changes such as declines in mean trophic level and body size truncation [42]. Evaluations of fisheries management measures in the East China Sea further suggest that persistent fishing pressure, together with fisheries management interventions, can jointly shape long-term changes in fish community structure [43]. Therefore, a more testable interpretation of the substantial contribution of the interannual trend is that conventional environmental gradients (temperature, salinity, depth, and Chl–a) remain insufficient to fully explain long-term community change. In this context, the temporal structure captured by this trend likely reflects additional long-term processes that are not represented by the current set of covariates. These processes may be jointly associated with persistent human disturbances (e.g., fishing pressure and changes in management measures) and broader environmental contexts such as climate warming, thereby driving sustained structural adjustments in fish communities [44]. However, their relative contributions and specific mechanisms still require quantitative testing in subsequent models by incorporating variables such as fishing pressure.

For the 12 focal fish species, the mean proportion of variation explained by random effects increased markedly (64.6%), and the spatial random effect became stronger (55.2%) (Table S4). This indicates that the screened set of focal species exhibits more pronounced spatial structuring; therefore, using these focal species as targets for monitoring or assessment is more conducive to highlighting community pattern information associated with spatial zonation and habitat related processes. Although random effects were dominant overall, the sources of variation still differed substantially among species, showing a differentiation into time dominated, space dominated, and mixed types. For example, *Cynoglossus robustus* exhibited a relatively low proportion of variation explained by random effects (20.4%), whereas the year trend was dominant (59.5%). This suggests that its occurrence is more likely influenced by long-term processes and can be classified as a time dominated type. *Cynoglossus robustus* is a benthic fish, and its distribution is closely associated with bottom habitats; therefore, its interannual variability may more readily reflect the combined effects of cumulative fishing pressure and long-term changes in benthic habitat conditions [45]. Previous studies have shown that bottom trawl fishing intensity in the East China Sea exhibits pronounced spatial concentration and interannual variability, and can exert persistent impacts on benthic ecosystems and benthic fish communities [46,47]. For *Miichthys miiuy*, the contributions of fixed and random effects were comparable, and the year trend accounted for a relatively high proportion (28.8%), indicating that it is both sensitive to changes in environmental conditions and influenced by long-term population processes; it can therefore be classified as a mixed type. *Miichthys miiuy* is an important commercial species in the Zhoushan Fishing Ground, and its spatial distribution is associated with water depth, benthic environments, and water-mass structure [48]. Its population fluctuations are likely related to recovery effects associated with management measures such as the summer fishing moratorium [43]. For the remaining species, such as *Larimichthys polyactis*, *Harpadon nehereus*, and *Coilia mystus*, random effects were dominant, suggesting that spatially structured processes not quantified and included in the model impose important constraints on their occurrence probability [19,21], and these species can be classified as space dominated types. Overall, these results indicate that the focal fish species set better reflects community patterns linked to spatial zonation and habitat related processes. The focal species can be classified into three types: time dominated, space dominated, and mixed. Building on this basis, identifying key habitats, assemblage structure, and dominant environmental drivers can provide more operational guidance for ecosystem-based fisheries management. Specifically, it can support the delineation of spatial management zones, help optimize the timing and areas of seasonal closures, and provide a basis for incorporating shared drivers into multispecies stock assessment and monitoring.

The variance partitioning above quantified the contributions of both fixed effects and random effects to variation in species occurrence probability. The following analyses focus on species level fixed effect significance, examining significant associations between environmental covariates and each focal species. Depth was significantly associated with occurrence probability for the largest number of species (5/12), whereas salinity was significant for the fewest (2/12). This suggests that, in the Zhoushan Fishing Ground during spring, differences in bottom habitat conditions represented by depth (including bottom hydrodynamics and mixing intensity, substrate characteristics, and near-bottom food availability) can jointly influence fishes’ energy acquisition and the costs of maintaining habitat use and persistence, as well as other related ecological aspects, thereby leading to spatially concordant changes in occurrence probability across multiple focal species. Because multiple focal species respond synchronously to depth, community composition is not simply the additive outcome of single species fluctuations, but instead manifests as a coordinated multispecies response to depth. In contrast, salinity showed separable significant effects for only a few focal species, which does not imply that salinity is unimportant, suggesting that its springtime variability is more likely to covary with gradients such as depth and temperature, making it difficult to generate a stable and consistent synchronous signal at the multispecies level [18,20]. Only one species showed overlapping significance for both the linear (SST) and quadratic (SST^2^) temperature terms: *Cynoglossus robustus* was positively associated with SST but negatively associated with SST^2^. This represents a typical unimodal response, whereby warming within a certain temperature range increases occurrence probability, whereas occurrence probability declines when temperatures deviate from the optimal range [49]. This may be related to the species’ relatively constrained preferred temperature window and benthic habitat specialization, making its occurrence more sensitive to deviations from suitable spring temperatures. Except for *Cynoglossus robustus*, only *Larimichthys polyactis* and *Lophius litulon* responded significantly to SST, whereas multiple species were significant only for the quadratic term (SST^2^). This indicates that, within the springtime temperature range observed, the effects of temperature on some focal species are more likely to be expressed through nonlinear components, such that occurrence probability is more sensitive to departures from suitable temperature conditions rather than showing a monotonic increase or decrease with temperature [21]. Including SST^2^ in the model captures such nonlinear temperature responses, enabling the temperature (SST and SST^2^) to detect significant associations with temperature across a larger number of species. Taken together, these results support Hypothesis 1, indicating that fish distributions are primarily structured by spatial processes and long-term temporal trends, whereas environmental covariates play a secondary yet species-specific role.

### 4.2. Focal Fish Species Form Two Distinct Assemblages with a Connector Species

We found that the 12 focal fish species could be identified as two assemblages, with strong positive residual correlations within each assemblage, predominantly negative residual correlations between assemblages, and one species exhibiting connecting characteristics (Figure 5). This pattern is analogous to ecological modularity in ecological network studies, where a network can be partitioned into several species modules that are weakly linked to each other, while species within each module are tightly connected. Modularity often implies that communities exhibit stable spatial zonation or niche differentiation, thereby enhancing overall ecosystem stability [50]. Assemblage 1 comprised *Larimichthys polyactis*, *Chelidonichthys kumu*, *Lophius litulon*, *Erisphex pottii*, *Thryssa kammalensis*, and *Cynoglossus robustus*. These species tend to occur in mid-water and benthic habitats with similar environmental conditions, and factors such as water mass structure, substrate properties, and food resources may constitute the environmental background driving their synchronous occurrence and synchronous absence [51]. Assemblage 2 consisted of *Coilia mystus*, *Cynoglossus lighti*, *Collichthys lucidus*, *Ctenotrypauchen chinensis*, and *Miichthys miiuy*, which typically occur in nearshore estuarine and high-productivity waters. In coastal estuarine areas, nutrient inputs, low salinity and highly turbid water masses, and other conditions may jointly shape their relatively consistent co-occurrence tendency [52]. The assemblages are more likely to reflect species’ shared preference for similar habitat conditions. Therefore, the negative associations between the two assemblages are more likely to arise from spatial separation of suitable habitats, with each assemblage being associated with different habitat conditions, resulting in predominantly negative correlations. Notably, *Harpadon nehereus* showed weak positive associations with species in Assemblage 1 and a weak negative association with *Collichthys lucidus* in Assemblage 2, exhibiting characteristics of a connector species. *Harpadon nehereus* shows relatively broad adaptability to water temperature and depth, is widely distributed in the Zhoushan Fishing Ground, and migrates from offshore to coastal waters for spawning each April [53]. Therefore, its occurrence and migration among different habitats increase the potential contact and co-occurrence probability between the two assemblages, thereby reducing their spatial segregation and reflecting its role as a connector species. Taken together, the structure comprising two assemblages and one connector species not only provides an ecological interpretation of community organization, but also offers an operational perspective for fisheries management that moves from single species to multispecies approaches. Under the multispecies mixed-fishery context of the Zhoushan Fishing Ground, implementing catch limits, gear regulations, or spatiotemporal controls on any one species within an assemblage may simultaneously alter fishing pressure and resource recovery for other species in the same assemblage [54]. The predominantly negative associations between assemblages point to habitat zonation, suggesting that management is better implemented and evaluated by habitat-based spatial units. The presence of a connector species suggests that zonation units are not completely isolated; because this species is associated with both assemblages, it should therefore be prioritized for monitoring. These patterns support Hypothesis 2, showing that after accounting for environmental differences, the community retains a stable residual organization that can be partitioned into two assemblages, with predominantly positive associations within each assemblage, predominantly negative associations between the assemblages, and one bridging species linking them.

### 4.3. Fish Distribution and Biomass Are Governed by Distinct Ecological Processes

In ecological terms, occurrence probability more likely reflects broad-scale habitat suitability and accessibility, as well as environmental tolerance, whereas biomass is further influenced by processes such as individual growth, feeding conditions, and intraspecific competition. In this study, we adopted a two-part hurdle framework by modeling species occurrence probability and positive biomass separately, which better matches the ecological processes of fish populations and can also alleviate the difficulties that zero inflation in fisheries data poses for parameter estimation and interpretation [31]. We found that for the same species, the direction and statistical support of responses to environmental covariates were not always fully consistent between the two stages. This pattern suggests that species occurrence and positive biomass conditional on occurrence may be governed by different ecological processes. The PA stage primarily characterizes whether a species can occur under given environmental conditions and therefore more readily reflects distribution patterns shaped by habitat suitability, environmental filtering, and spatially structured processes. In contrast, the POSBIO stage quantifies local positive biomass conditional on occurrence, which is often jointly regulated by resource availability, individual growth and aggregation behavior, and intraspecific competition. In a practical fisheries context, the two-part framework separates distribution probability from positive biomass, thereby providing a basis for more targeted fisheries management.

For example, *Harpadon nehereus* was significantly positively associated with SST^2^ in the PA model but significantly negatively associated with SST^2^ in the POSBIO model, suggesting that increasing temperature exerts opposite effects on its species occurrence probability and positive biomass. On the one hand, temperature increases may allow more areas to fall within its thermal tolerance window, thereby increasing its occurrence probability; on the other hand, as temperature increases and deviates from its optimal thermal niche range, higher metabolic costs and intensified interspecific competition may constrain the formation of higher biomass in the corresponding areas [49]. Therefore, an increase in species occurrence probability does not necessarily imply an increase in positive biomass; in management, resource recovery should not be inferred solely from an increase in species occurrence probability, nor should fishing intensity be increased on this basis. Similarly, *Miichthys miiuy* showed a significant positive association with depth in the POSBIO model but a significant negative association in the PA model, indicating a higher occurrence probability in shallow waters, whereas its positive biomass is higher in deeper waters. For *Miichthys miiuy*, nearshore shallow waters, shaped by the combined effects of terrestrial inputs and hydrodynamic disturbance, likely provide higher food resource availability and greater habitat heterogeneity, thereby increasing its occurrence probability. In contrast, deeper waters may offer more stable bottom environmental conditions and, under the combined influence of water mass structure and the spatial availability of key prey resources, may provide more favorable conditions for residence and aggregation, resulting in higher positive biomass [20,31]. These results suggest that spatial fisheries management and conservation measures should distinguish between areas with high occurrence probability and those with high biomass. In contrast, *Larimichthys polyactis*, *Lophius litulon*, and *Cynoglossus robustus* showed consistently significant positive associations with depth in both stages, suggesting that protecting benthic habitats may simultaneously support the maintenance of their distributions and the recovery of their resources. For example, protection of benthic habitats is closely linked to the quality of nursery habitats, and high-quality nursery habitats can improve juvenile growth conditions and increase early survival and recruitment success, thereby helping to maintain population distributions and promote resource recovery over longer timescales. Two-part hurdle modeling has been applied in survey and assessment studies in regions such as the North Sea and the Baltic Sea and has demonstrated good applicability [31,55,56]. Specifically, these studies used two-part hurdle models to distinguish occurrence from either positive CPUE or positive biomass, thereby supporting the prediction of marine fish biomass distributions, ecological model construction, and the establishment of resource monitoring indicators. By decomposing the process of species occurrence probability and positive biomass for fishes in the Zhoushan Fishing Ground, this study provides management relevant evidence at both the distribution and biomass levels. Specifically, the PA component can be used to delineate suitable habitats and potential expansion areas, whereas the POSBIO component can be used to identify high biomass aggregation areas and to inform the establishment of spatially targeted harvest controls and precautionary limits on fishing intensity. Together, these complementary outputs support differentiated management actions. Overall, these findings support Hypothesis 3, showing that species occurrence and positive biomass are governed by different ecological processes, and that their responses to the same environmental covariates can differ.

## 5. Conclusions

Based on spring bottom-trawl survey data from the Zhoushan Fishing Ground collected during 2014–2024, together with environmental data, we developed a two-part hurdle model within the HMSC framework. The results showed that variation in fish occurrence probability was dominated by the spatial random effect, with concurrent interannual trends, indicating that unobserved spatially structured processes and long-term changes jointly shape community patterns. Among the environmental effects, depth was significant for a relatively larger subset of species, whereas the independent signal of salinity was comparatively weak. Residual correlations further indicated that the focal fish species could be partitioned into two major assemblages and a connector species by decomposing “occurrence” and “intensity” under the two-part hurdle model framework. These findings provide a regulatory basis for the Zhoushan Fishing Ground that is more consistent with actual fisheries processes. This study relied only on spring survey data, making it difficult to characterize seasonal variability and dynamics throughout the year. Future research should be extended to multiple seasons and should systematically incorporate indicators of human disturbance, such as fishing intensity and fishing moratoria, to further improve the quantitative explanatory capacity for long-term change and human impacts, ultimately enhancing the applicability of the findings to fisheries resource assessment and management in the Zhoushan Fishing Ground.

## Figures and Tables

**Table 1:** 12 focal fish species in the Zhoushan Fishing Ground.

Number	Scientific Name
S1	*Harpadon nehereus*
S2	*Cynoglossus lighti*
S3	*Coilia mystus*
S4	*Collichthys lucidus*
S5	*Larimichthys polyactis*
S6	*Chelidonichthys kumu*
S7	*Lophius litulon*
S8	*Erisphex pottii*
S9	*Ctenotrypauchen chinensis*
S10	*Thryssa kammalensis*
S11	*Miichthys miiuy*
S12	*Cynoglossus robustus*

**Table 2:** Results of model convergence diagnostics and performance evaluation.

Indicator Category	Metric	PA (Mean)	POSBIO (Mean)
Convergence diagnostics	PSRF (β)	1.00	1.00
	PSRF (γ)	1.00	1.00
	ESS (β)	5983.13	8525.26
	ESS (γ)	7318.55	7492.74
Model performance (in-sample)	AUC	0.84	—
	Tjur’s R^2^	0.30	—
	RMSE	—	1.04
	R^2^	—	0.25
Model performance (fivefold cross validation)	AUC	0.81	—
	Tjur’s R^2^	0.28	—
	RMSE	—	1.11
	R^2^	—	0.07

## Data Availability

The original contributions presented in the study are included in the article. Further inquiries can be directed to the corresponding author.

## Supplementary Materials

The following supporting information can be downloaded at: https://www.mdpi.com/article/10.3390/ani16060865/s1, Table S1: Environmental covariate data product information and processing details; Table S2: List of 26 species used for model fitting; Table S3: Variance partitioning details for focal fish species in the PA model; Table S4: Variance partitioning of occurrence probability for the 12 focal fish species in the PA model.
