# Supplementary material for: Environmental Responses and Interspecific Associations of Fish Communities in the Zhoushan Fishing Ground Revealed by HMSC
Source: Animals (Basel). 2026 Mar 10;16(6):865. doi: 10.3390/ani16060865 (PMC13023308; doi:10.3390/ani16060865)
Supplement: Supplementary file 1 [file animals-16-00865-s001.zip › animals-4165623-supplementary.pdf]

Table S1. Environmental covariate data product information and processing details.

Item	SST	Chl-a	SSS
Dataset name	Global Ocean OSTIA Sea Surface Temperature and Sea Ice Analysis	Global Ocean Colour (Copernicus-GlobColour), Bio-Geo-Chemical, L4 (monthly and interpolated) from Satellite Observations (1997-ongoing)	Multi Observation Global Ocean Sea Surface Salinity and Sea Surface Density
Product ID	SST_GLO_SST_L4_NRT_OBSERVATIONS_010_001	OCEANCOLOUR_GLO_BGC_L4_MY_009_104	MULTIOBS_GLO_PHY_S_SURFACE_MYNRT_015_013
DOI	https://doi.org/10.48670/moi-00165	https://doi.org/10.48670/moi-00281	https://doi.org/10.48670/moi-00051
Spatial resolution	0.05° × 0.05°	4 × 4 km	0.125° × 0.125°
Temporal resolution	Daily	Monthly	Monthly
Temporal aggregation strategy relative to sampling dates	Environmental covariates were defined as the April mean for each fisheries resource survey year. Specifically, SST was aggregated from daily values to the April monthly mean, whereas SSS and Chl-a were extracted directly from monthly products, with no additional temporal aggregation.		
Spatial extraction method and rationale	Nearest-neighbor extraction at station coordinates (no spatial interpolation) was applied to extract environmental values at each sampling station, thereby preserving native grid values, avoiding artificial smoothing, and ensuring fully reproducible extraction across different data products.		

Table S2. List of 26 species used for model fitting

Number	Scientific Name
S1	*Harpadon nehereus*
S2	*Cynoglossus lighti*
S3	*Coilia mystus*
S4	*Collichthys lucidus*
S5	*Larimichthys polyactis*
S6	*Chelidonichthys kumu*
S7	*Lophius litulon*
S8	*Erisphex pottii*
S9	*Ctenotrypauchen chinensis*
S10	*Thryssa kammalensis*
S11	*Miichthys miiuy*
S12	*Cynoglossus robustus*
S13	*Odontamblyopus rubicundus*
S14	*Coilia nasus*
S15	*Chaemrichthys stigmatias*
S16	*Trichiurus lepturus*
S17	*Cynoglossus purpureomaculatus*
S18	*Cynoglossus interruptus*
S19	*Setipinna taty*
S20	*Benthosema pterotum*
S21	*Muraenesox cinereus*
S22	*Pampus argenteus*
S23	*Sebastiscus marmoratus*
S24	*Johnius belangerii*

S25	*Chaeturichthys hexanema*
S26	*Corythoichthys schultzi*

Table S3. Variance partitioning details for focal fish species in the PA model

Species	Group	Type	Value
Harpadon nehereus	Temperature	Fixed	0.040
	Chl–a	Fixed	0.012
	SSS	Fixed	0.039
	Depth	Fixed	0.021
	Year trend	Fixed	0.053
	Site	Random	0.717
	Year	Random	0.119
	Random	Random	0.835
Cynoglossus lighti	Temperature	Fixed	0.054
	Chl–a	Fixed	0.052
	SSS	Fixed	0.012
	Depth	Fixed	0.009
	Year trend	Fixed	0.193
	Site	Random	0.455
	Year	Random	0.225
	Random	Random	0.680
Coilia mystus	Temperature	Fixed	0.035
	Chl–a	Fixed	0.025
	SSS	Fixed	0.080
	Depth	Fixed	0.019
	Year trend	Fixed	0.152
	Site	Random	0.653
	Year	Random	0.034
	Random	Random	0.688
Collichthys lucidus	Temperature	Fixed	0.027
	Chl–a	Fixed	0.049
	SSS	Fixed	0.065
	Depth	Fixed	0.015
	Year trend	Fixed	0.050
	Site	Random	0.777
	Year	Random	0.017
	Random	Random	0.794
Larimichthys polyactis	Temperature	Fixed	0.112
	Chl–a	Fixed	0.013
	SSS	Fixed	0.008
	Depth	Fixed	0.066
	Year trend	Fixed	0.181
	Site	Random	0.610
	Year	Random	0.011
	Random	Random	0.621
Chelidonichthys kumu	Temperature	Fixed	0.155
	Chl–a	Fixed	0.092
	SSS	Fixed	0.014
	Depth	Fixed	0.020
	Year trend	Fixed	0.061
	Site	Random	0.374
	Year	Random	0.284

	Random	Random	0.658
Lophius litulon	Temperature	Fixed	0.191
	Chl–a	Fixed	0.018
	SSS	Fixed	0.006
	Depth	Fixed	0.009
	Year trend	Fixed	0.163
	Site	Random	0.495
	Year	Random	0.118
	Random	Random	0.612
Erisphex pottii	Temperature	Fixed	0.064
	Chl–a	Fixed	0.017
	SSS	Fixed	0.010
	Depth	Fixed	0.092
	Year trend	Fixed	0.094
	Site	Random	0.544
	Year	Random	0.178
	Random	Random	0.722
Ctenotrypauchen chinensis	Temperature	Fixed	0.070
	Chl–a	Fixed	0.102
	SSS	Fixed	0.025
	Depth	Fixed	0.116
	Year trend	Fixed	0.122
	Site	Random	0.531
	Year	Random	0.035
	Random	Random	0.565
Thryssa kammalensis	Temperature	Fixed	0.062
	Chl–a	Fixed	0.024
	SSS	Fixed	0.007
	Depth	Fixed	0.007
	Year trend	Fixed	0.026
	Site	Random	0.814
	Year	Random	0.060
	Random	Random	0.874
Miichthys miiuy	Temperature	Fixed	0.087
	Chl–a	Fixed	0.034
	SSS	Fixed	0.044
	Depth	Fixed	0.044
	Year trend	Fixed	0.288
	Site	Random	0.471
	Year	Random	0.032
	Random	Random	0.503
Cynoglossus robustus	Temperature	Fixed	0.081
	Chl–a	Fixed	0.020
	SSS	Fixed	0.079
	Depth	Fixed	0.020
	Year trend	Fixed	0.595
	Site	Random	0.186
	Year	Random	0.017
	Random	Random	0.204

Table S4. Variance partitioning of occurrence probability for the 12 focal fish species in the PA model.

Group	Type	Mean
Temperature	Fixed	0.082
Chl–a	Fixed	0.038
SSS	Fixed	0.032
Depth	Fixed	0.037
Year trend	Fixed	0.165
Random	Random	0.646
Site	Random	0.552
Year	Random	0.094
